# Supplementary material for: Consensus recommendations for hyperpolarized [1‐ 13C]pyruvate MRI multi‐center human studies
Source: Magn Reson Med. 2025 Jun 16;94(4):1386–400. doi: 10.1002/mrm.30570 (PMC12236423; doi:10.1002/mrm.30570)
Supplement: Supplementary file 1 — Data S1. Supporting Information. [file MRM-94-1386-s001.docx]

Supplementary material for “Consensus Recommendations for Hyperpolarized [1‐^13^C]pyruvate MRI Multi-center Human Studies”

The supplementary material includes additional details from the consensus process such as all statements posed to the members, the detailed results of scoring, and information about the participants.

Tables:

1. Information about all group members who participated.
2. Listing of all statements along with a summary of round 2 scoring indicating the most common outcome (Disagreement/Uncertain/Agreement) and whether consensus was reached (Consensus/No consensus).
3. Summary of statements scoring for Round 1 and Round 2.
4. Statements altered between round 1 and round 2.

***Supplementary Table S1****: Group Members participating in the consensus voting (name, speciality, country/site, years of C13 expertise). Panellist with * contributed to round 1 scoring but were not able to participate in round 2 face-to-face meeting.*

| Name | Specialty | Country/site | Years of expertise |
| --- | --- | --- | --- |
| Esben Sovso Szocska | Engineering / Physics | Aarhus University | 10 |
| Chris Laustsen | Engineering / Physics, Pharmacy Production & Manufacturing | Aarhus University | 15 |
| Lotte Bonde Bertelsen | Engineering / Physics, Pharmacy Production & Manufacturing | Aarhus University | 10 |
| Ferdia Gallagher | Clinician | Cambridge University | 18 |
| Ashley Grimmer | Pharmacy Production & Manufacturing | Cambridge University | 6.5 |
| Mary McLean | Engineering / Physics | Cambridge University | 10 |
| Ching-Yi Hsieh | Engineering / Physics | Chang Gung University and Chang Gung Memorial Hospital at Linkou, Taiwan | 6 |
| Sebastian Kozerke * | Engineering / Physics | ETH Zurich | 15 |
| Albert Chen * | Engineering / Physics | GE Healthcare | 20 |
| Adam Gaunt | Engineering / Physics, Other | GE Healthcare | 10 |
| Arnaud Comment | Engineering / Physics, Pharmacy Production & Manufacturing | GE Healthcare | 15 |
| Jim Bankson | Engineering / Physics | MD Anderson Cancer Center | 14 |
| Kayvan Keshari | Engineering / Physics | Memorial Sloan Kettering - Cancer Center | 15 |
| James Grist | Engineering / Physics | Nottingham University & Oxford University | 10 |
| Damian Tyler | Engineering / Physics | Oxford University | 19 |
| Fulvio Zaccagna | Radiologist / Radiographer, Clinician | Oxford University & Cambridge University | 9 |
| Titus Lanz * | Engineering / Physics | RAPID Biomedical GmbH | 20 |
| Jan Henrik Ardenkjær-Larsen | Engineering / Physics | Technical University of Denmark | 25 |
| Mathilde Lerche | Engineering / Physics, Other | Technical University of Denmark | 22 |
| Peder Larson | Engineering / Physics | UCSF | 17 |
| Duan Xu | Engineering / Physics | UCSF | 13 |
| Bob Bok | Clinician | UCSF | 17 |
| Jim Slater | Pharmacy Production & Manufacturing | UCSF | 8 |
| Duy Dang | Pharmacy Production & Manufacturing | UCSF | 2 |
| Adam Autry | Engineering / Physics | UCSF | 8 |
| Rafat Chowdhury | Engineering / Physics | University College London | 7 |
| Richard Hesketh | Radiologist / Radiographer, Clinician | University College London | 6 |
| Shonit Punwani | Engineering / Physics, Radiologist / Radiographer | University College London | 9 |
| Dirk Mayer | Engineering / Physics | University of Maryland | 17 |
| Chuck Cunningham | Engineering / Physics | University of Toronto | 20 |
| Jae Mo Park | Engineering / Physics | UT Southwestern | 14 |
| Vlad Zaha | Radiologist / Radiographer, Clinician | UT Southwestern | 6 |

**Supplementary Table S2**: Summary of round 2 scoring for all statements included. The outcome category (1-3 disagree, 4-6 uncertain or 7-9 agree) was defined by the median score across all non-zero responders. Consensus indicates whether a 66% or more of the non-zero responses were in the outcome category when at least 14 votes were made.

| No. | Statement | Outcome  (round 2) | Consensus (round 2) | Fraction | Percentage |
| --- | --- | --- | --- | --- | --- |
| 1A-1: For multi-center studies: | |  |  |  |  |
| 1 | a. all sites have to follow the same 13C pyruvate preparation guidelines (e.g. Sterile Compounding or Terminal Sterilization). | Disagreement | Consensus | 17/25 | 68% |
| 2 | b. it is preferred that all sites follow the same 13C pyruvate preparation guidelines. | Agreement | No consensus | 15/25 | 60% |
| 1A-2: For multi-center studies: | |  |  |  |  |
| 3 | a. all sites have to follow the same SOPs for 13C pyruvate preparation process. | Disagreement | Consensus | 20/26 | 77% |
| 4 | b. it is preferred that all sites follow the same SOPs for 13C pyruvate preparation process. | Agreement | No consensus | 15/26 | 58% |
| 1A-3: For multi-center studies: | |  |  |  |  |
| 5 | a. all sites have to have the same environment and facilities (e.g. clean room, isolator, clean bench) for 13C pyruvate preparation process. | Disagreement | Consensus | 19/26 | 73% |
| 6 | b. it is preferred that all sites have the same environment and facilities for 13C pyruvate preparation process. | Agreement | No consensus | 13/26 | 50% |
| 1B-1: For multi-center studies: | |  |  |  |  |
| 7 | a. all sites have to follow the same release criteria for release of hyperpolarized 13C pyruvate. | Agreement | Consensus | 21/26 | 81% |
| 8 | b. it is preferred that all sites follow the same release criteria for release of hyperpolarized 13C pyruvate. | Agreement | Consensus | 25/26 | 96% |
| 1B-2: The following have to be part of the dose release criteria: | |  |  |  |  |
| 9 | a. PA concentration | Agreement | Consensus | 25/26 | 96% |
| 10 | b. EPA concentration | Agreement | Consensus | 26/26 | 100% |
| 11 | c. pH | Agreement | Consensus | 26/26 | 100% |
| 12 | d. Temperature | Agreement | Consensus | 23/26 | 88% |
| 13 | e. Volume | Agreement | Consensus | 21/26 | 81% |
| 14 | f. Polarization | Agreement | No consensus | 14/25 | 56% |
| 1B-3: For multi-center studies: | |  |  |  |  |
| 15 | a. all sites have to use the same QC system measurement for dose release. | Disagreement | Consensus | 17/24 | 71% |
| 16 | b. it is preferred that all sites use the same QC system measurement for dose release. | Uncertain | No consensus | 7/26 | 27% |
| 2A-1: For multi-center studies: | |  |  |  |  |
| 17 | a. all sites have to use a scanner from the same manufacturer. (If you are a direct employee of any of the manufacturers listed below, please select “0”) | Disagreement | Consensus | 19/22 | 86% |
| 18 | b. it is preferred that all sites use a scanner from the same manufacturer. | Disagreement | Consensus | 17/23 | 74% |
| 2A-2: Viable options for participation in multi-center studies are: (If you are a direct employee of any of the manufacturers listed below, please select “0”) | | | | |  |
| 19 | a. GE | Agreement | Consensus | 22/22 | 100% |
| 20 | b. Siemens | Agreement | Consensus | 21/22 | 95% |
| 21 | c. Phillips | Agreement | Consensus | 17/19 | 89% |
| 2A-3: Preferred manufacturer is: (If you are a direct employee of any of the manufacturers listed below, please select “0”) | | |  |  |  |
| 22 | a. GE | Agreement | No consensus | 13/20 | 65% |
| 23 | b. Siemens | Uncertain | No consensus | 10/20 | 50% |
| 24 | c. Phillips | Uncertain | No consensus | 10/19 | 53% |
| 2A-4: For multi-center studies: | |  |  |  |  |
| 25 | a. the magnet field strength B0 has to be the same for all sites. | Agreement | Consensus | 19/24 | 79% |
| 26 | b. it is preferred that all sites use a scanner with the same B0. | Agreement | Consensus | 23/24 | 96% |
| 2A-5: Viable options for participation in multi-center study are: | | *Question was removed* | | | |
| 27 | a. 0.5T |  |  |  |  |
| 28 | b. 1.5T |  |  |  |  |
| 29 | c. 3T |  |  |  |  |
| 30 | d. 7T |  |  |  |  |
| 2A-6: Preferred B0 is: | |  |  |  |  |
| 31 | a. 0.5T | Disagreement | Consensus | 19/23 | 83% |
| 32 | b. 1.5T | Uncertain | Consensus | 16/24 | 67% |
| 33 | c. 3T | Agreement | Consensus | 23/24 | 96% |
| 34 | d. 7T | Uncertain | No consensus | 11/24 | 46% |
| 2A-7: For multi-center studies: | |  |  |  |  |
| 35 | a. all sites have to have the same maximum gradient strength. | Agreement | No consensus | 13/23 | 57% |
| 36 | b. it is preferred that all sites have the same maximum gradient strength. | Agreement | Consensus | 22/23 | 96% |
| 2A-8: For multi-center studies: | |  |  |  |  |
| 37 | a. all sites have to have the same maximum gradient slew rate. | Agreement | No consensus | 14/23 | 61% |
| 38 | b. it is preferred that all sites have the same maximum gradient slew rate. | Agreement | Consensus | 22/23 | 96% |
| 2A-9: For multi-center studies: | |  |  |  |  |
| 39 | a. all sites have to have the same maximum available RF amplifier power. | Agreement | No consensus | 12/23 | 52% |
| 40 | b. it is preferred that all sites have the same maximum available amplifier power. | Agreement | Consensus | 22/23 | 96% |
| 2A-10: For multi-center studies: | |  |  |  |  |
| 41 | a. all sites have to have the same manufacturer and model for 13C transmit and receive coils. | Disagreement | No consensus | 13/24 | 54% |
| 42 | b. it is preferred that all sites have the same manufacturer and model for 13C transmit and receive coils. | Agreement | Consensus | 20/24 | 83% |
| 2A-11: For multi-center studies: | |  |  |  |  |
| 43 | a. all sites have to have the same fundamental geometry design for 13C transmit and receive coils. | Uncertain | No consensus | 6/23 | 26% |
| 44 | b. it is preferred that all sites have the same fundamental geometry design for 13C transmit and receive coils. | Agreement | Consensus | 22/23 | 96% |
| 2A-12: For multi-center studies: | |  |  |  |  |
| 45 | a. all sites have to have a reference phantom in the field of view of 13C coil. | Uncertain | No consensus | 8/24 | 33% |
| 46 | b. it is preferred that all sites have to have a reference phantom in the field of view of 13C coil. | Agreement | No consensus | 15/24 | 63% |
| 2A-13: For multi-center studies: | |  |  |  |  |
| 47 | a. all sites have to be able to acquire 1H and 13C images without repositioning the patient in between acquisition scans. | Agreement | Consensus | 16/24 | 67% |
| 48 | b. it is preferred that all sites are able to acquire 1H and 13C images without repositioning the patient in between acquisition scans. | Agreement | Consensus | 23/24 | 96% |
| 2B-1: For multi-center studies: | |  |  |  |  |
| 49 | a. all sites have to perform an agreed-upon scanner site qualification (e.g. QC experiments). | Agreement | Consensus | 23/24 | 96% |
| 50 | b. it is preferred that all sites perform an agreed-upon scanner site qualification. | Agreement | Consensus | 24/24 | 100% |
| 2B-2: Viable approaches to scanner site qualification include use of: | |  |  |  |  |
| 51 | a. Static thermal phantoms | Agreement | Consensus | 25/25 | 100% |
| 52 | b. Dynamic HP phantoms (e.g. with enzymatic conversion) | Uncertain | No consensus | 5/25 | 20% |
| 53 | c. Travelling humans injected with HP 13C | Uncertain | No consensus | 8/24 | 33% |
| 2B-3: Preferred approach is: | |  |  |  |  |
| 54 | a. Static thermal phantoms | Agreement | Consensus | 25/25 | 100% |
| 55 | b. Dynamic HP phantoms (e.g. with enzymatic conversion) | Uncertain | No consensus | 11/25 | 44% |
| 56 | c. Travelling humans injected with HP 13C | Disagreement | No consensus | 12/24 | 50% |
| 2B-4: For multi-center studies: | |  |  |  |  |
| 57 | a. all sites have to perform on-going hardware quality assurance throughout the study. | Agreement | Consensus | 20/25 | 80% |
| 58 | b. it is preferred that all sites perform on-going hardware quality assurance throughout the study. | Agreement | Consensus | 24/25 | 96% |
| 2C-1: For multi-center studies: | |  |  |  |  |
| 59 | a. all sites have to follow the same protocol for adjustment of RF pulse power. | Disagreement | No consensus | 11/22 | 50% |
| 60 | b. it is preferred that all sites follow the same protocol for adjustment of RF pulse power. | Agreement | Consensus | 17/22 | 77% |
| 2C-2: Viable options for pre-scan setting of RF pulse power are: | |  |  |  |  |
| 61 | a. Automated optimization, e.g. Bloch-Siegert | Agreement | Consensus | 21/22 | 95% |
| 62 | b. Manual optimization, e.g. power sweep | Agreement | Consensus | 22/22 | 100% |
| 63 | c. Predetermined values from phantom calibrations | Agreement | No consensus | 13/22 | 59% |
| 2C-3: Preferred method of adjustment of RF pulse power: | |  |  |  |  |
| 64 | a. Automated optimization, e.g. Bloch-Siegert | Agreement | Consensus | 21/21 | 100% |
| 65 | b. Manual optimization, e.g. power sweep | Agreement | Consensus | 19/21 | 90% |
| 66 | c. Predetermined values from phantom calibrations | Uncertain | No consensus | 7/21 | 33% |
| 2C-4: For multi-center studies: | |  |  |  |  |
| 67 | a. all sites have to follow the same protocol for pre-scan adjustment of center frequency. | Uncertain | No consensus | 4/24 | 17% |
| 68 | b. it is preferred that all sites follow the same protocol for pre-scan adjustment of center frequency. | Agreement | Consensus | 23/24 | 96% |
| 2C-5: Viable options for pre-scan setting of center frequency are: | |  |  |  |  |
| 69 | a. 1H frequency in tissue | Agreement | Consensus | 24/24 | 100% |
| 70 | b. 13C frequency in phantom | Agreement | Consensus | 17/24 | 71% |
| 71 | c. 13C frequency in tissue | Uncertain | No consensus | 11/24 | 46% |
| 72 | d. 23Na frequency in tissue | Uncertain | No consensus | 12/24 | 50% |
| 2C-6: Preferred method of adjustment of center frequency is: | |  |  |  |  |
| 73 | a. 1H frequency in tissue | Agreement | Consensus | 23/24 | 96% |
| 74 | b. 13C frequency in phantom | Uncertain | No consensus | 9/24 | 38% |
| 75 | c. 13C frequency in tissue | Uncertain | No consensus | 10/24 | 42% |
| 76 | d. 23Na frequency in tissue | Uncertain | No consensus | 11/24 | 46% |
| 2C-7: For multi-center studies: | |  |  |  |  |
| 77 | a. all sites have to follow the same protocol for adjustment of receive gains. | Uncertain | No consensus | 7/21 | 33% |
| 78 | b. it is preferred that all sites follow the same protocol for adjustment of receive gains. | Uncertain | No consensus | 6/21 | 29% |
| 2C-8: Viable options for pre-scan setting of receive gains are: | |  |  |  |  |
| 79 | a. Maximum values possible | Disagreement | No consensus | 11/21 | 52% |
| 80 | b. Based on previous in vivo measurements | Agreement | Consensus | 19/22 | 86% |
| 81 | c. Based on phantom measurements | Agreement | No consensus | 12/22 | 55% |
| 2C-9: Preferred method of adjustment of receive gains is: | |  |  |  |  |
| 82 | a. Maximum values possible | Disagreement | No consensus | 11/21 | 52% |
| 83 | b. Based on previous in vivo measurements | Agreement | Consensus | 19/22 | 86% |
| 84 | c. Based on phantom measurements | Agreement | No consensus | 12/22 | 55% |
| 2C-10: For multi-center studies: | |  |  |  |  |
| 85 | a. all sites have to follow the same protocol for shimming to optimize B0 homogeneity. | Uncertain | No consensus | 7/24 | 29% |
| 86 | b. it is preferred that all sites follow the same protocol for shimming to optimize B0 homogeneity. | Agreement | Consensus | 21/24 | 88% |
| 2C-11: Viable options for shimming are: | |  |  |  |  |
| 87 | a. Automated shimming over imaging volume | Agreement | Consensus | 22/23 | 96% |
| 88 | b. Automated shimming over a ROI (e.g. PRESS Box) | Agreement | Consensus | 21/23 | 91% |
| 89 | c. Manual shimming over imaging volume | Uncertain | No consensus | 6/23 | 26% |
| 90 | d. Manual shimming over a ROI | Agreement | No consensus | 12/23 | 52% |
| 2C-12: Preferred methods of adjustment of shims are: | |  |  |  |  |
| 91 | a. Automated shimming over imaging volume | Agreement | Consensus | 22/23 | 96% |
| 92 | b. Automated shimming over a ROI (e.g. PRESS Box) | Agreement | Consensus | 22/23 | 96% |
| 93 | c. Manual shimming over imaging volume | Uncertain | No consensus | 11/23 | 48% |
| 94 | d. Manual shimming over a ROI | Uncertain | No consensus | 10/23 | 43% |
| 2C-13: For multi-center studies: | |  |  |  |  |
| 95 | a. all sites have to perform higher-order shimming. | Disagreement | No consensus | 15/24 | 63% |
| 96 | b. it is preferred that all sites perform higher-order shimming. | Uncertain | No consensus | 6/24 | 25% |
| 3A-1: For multi-center studies: | |  |  |  |  |
| 97 | a. all sites have to use the same pulse sequence type (e.g. MRS/I, metabolite-specific imaging, or chemical shift encoding) when aggregating data across sites. | Uncertain | No consensus | 10/26 | 38% |
| 98 | b. it is preferred that all sites use the same pulse sequence type when aggregating data across sites. | Agreement | Consensus | 25/26 | 96% |
| 3A-2: For multi-center studies: | |  |  |  |  |
| 99 | a. all sites have to use sequences that provide spatial localization. | Agreement | No consensus | 14/26 | 54% |
| 100 | b. it is preferred that all sites use sequences that provide spatial localization. | Agreement | Consensus | 22/26 | 85% |
| 3A-3: For multi-center studies: | |  |  |  |  |
| 101 | a. all sites have to use sequences that provide spectral information (e.g. generate metabolite maps, provide a spectrum). | Uncertain | No consensus | 9/26 | 35% |
| 102 | b. it is preferred that all sites use sequences that provide spectral information (e.g. generate metabolite maps, provide a spectrum). | Agreement | Consensus | 26/26 | 100% |
| 3A-4: For multi-center studies: | |  |  |  |  |
| 103 | a. all sites have to use sequences that provide dynamic (time resolved) data. | Uncertain | No consensus | 8/26 | 31% |
| 104 | b. it is preferred that all sites use sequences that provide dynamic (time resolved) data. | Agreement | Consensus | 24/26 | 92% |
| 3A-5: For multi-center studies: | |  |  |  |  |
| 105 | a. all sites have to capture the bolus of pyruvate in the data acquisition. | Uncertain | No consensus | 11/26 | 42% |
| 106 | b. it is preferred that all sites capture the bolus of pyruvate in the data acquisition. | Agreement | Consensus | 23/26 | 88% |
| 3A-6: For a given pulse sequence: | |  |  |  |  |
| 107 | a. all sites have to use the same flip angle scheme. | Uncertain | No consensus | 4/26 | 15% |
| 108 | b. it is preferred that all sites use the same flip angle scheme. | Agreement | Consensus | 25/26 | 96% |
| 3A-7: For multi-center studies: | |  |  |  |  |
| 109 | a. all sites have to use the same spatial resolution when aggregating data across sites. | Uncertain | No consensus | 9/26 | 35% |
| 110 | b. it is preferred that all sites use the same spatial resolution when aggregating data across sites. | Agreement | Consensus | 25/26 | 96% |
| 3A-8: For multi-center studies: | |  |  |  |  |
| 111 | a. all sites have to have the same timing parameters (start time, temporal resolution, and number of timeframes) when aggregating data across different sites. | Uncertain | No consensus | 7/26 | 27% |
| 112 | b. it is preferred that all sites have the same timing parameters when aggregating data across different sites. | Agreement | Consensus | 25/26 | 96% |
| 3A-9: For multi-center studies: | |  |  |  |  |
| 113 | a. all sites have to use the same undersampling method (e.g. partial Fourier acceleration, parallel imaging acceleration, model-based acceleration methods such as compressed sensing, low-rank reconstructions, and deep learning). | Uncertain | No consensus | 4/23 | 17% |
| 114 | b. it is preferred that all sites have the same undersampling method (e.g. partial Fourier acceleration, parallel imaging acceleration, model-based acceleration methods such as compressed sensing, low-rank reconstructions, and deep learning). | Agreement | Consensus | 22/23 | 96% |
| 3A-10: Best practice recommendation | |  |  |  |  |
| 115 | a. Any contrast injection needed for 1H MRI have to be performed after the HP 13C study. | Agreement | Consensus | 24/25 | 96% |
| 116 | b. 1H images with coverage equal to or exceeding the 13C FOV have to be acquired, for anatomic reference. | Agreement | Consensus | 26/26 | 100% |
| 117 | c. Acquire a B0 field map to identify and potentially correct for artifacts caused by B0 inhomogeneity | Agreement | Consensus | 25/26 | 96% |
| 3B-1: Best practice recommendation for PROSTATE acquisition includes: | |  |  |  |  |
| 118 | a. Multi-shot readouts | Uncertain | No consensus | 7/15 | 47% |
| 119 | b. Single-shot readouts | Agreement | No consensus | 8/15 | 53% |
| 120 | c. Metabolite-specific RF excitation | Agreement | Consensus | 13/15 | 87% |
| 121 | d. Resolve a spectrum | Agreement | Consensus | 13/15 | 87% |
| 122 | e. Respiratory gating | Disagreement | Consensus | 13/15 | 87% |
| 123 | f. Cardiac gating | Disagreement | Consensus | 14/15 | 93% |
| 3B-2: Best practice recommendation for BRAIN acquisition includes: | |  |  |  |  |
| 124 | a. Multi-shot readouts | Uncertain | No consensus | 7/19 | 37% |
| 125 | b. Single-shot readouts | Agreement | Consensus | 14/19 | 74% |
| 126 | c. Metabolite-specific RF excitation | Agreement | Consensus | 17/19 | 89% |
| 127 | d. Resolve a spectrum | Agreement | Consensus | 14/19 | 74% |
| 128 | e. Respiratory gating | Disagreement | Consensus | 18/20 | 90% |
| 129 | f. Cardiac gating | Disagreement | Consensus | 18/20 | 90% |
| 3B-3: Best practice recommendation for CARDIAC acquisition includes: | |  |  |  |  |
| 130 | a. Multi-shot readouts | Disagreement | No consensus | 8/14 | 57% |
| 131 | b. Single-shot readouts | Agreement | Consensus | 12/14 | 86% |
| 132 | c. Metabolite-specific RF excitation | Agreement | Consensus | 12/14 | 86% |
| 133 | d. Resolve a spectrum | Agreement | Consensus | 10/14 | 71% |
| 134 | e. Respiratory gating | Agreement | Consensus | 13/15 | 87% |
| 135 | f. Cardiac gating | Agreement | Consensus | 14/15 | 93% |
| 3B-4: Best practice recommendation for ABDOMEN acquisition includes: | |  |  |  |  |
| 136 | a. Multi-shot readouts | Uncertain | No consensus | 10/19 | 53% |
| 137 | b. Single-shot readouts | Agreement | Consensus | 13/19 | 68% |
| 138 | c. Metabolite-specific RF excitation | Agreement | Consensus | 17/20 | 85% |
| 139 | d. Resolve a spectrum | Agreement | Consensus | 15/20 | 75% |
| 140 | e. Respiratory gating | Agreement | Consensus | 18/20 | 90% |
| 141 | f. Cardiac gating | Uncertain | No consensus | 8/20 | 40% |
| 3C-1: For multi-center studies: | |  |  |  |  |
| 142 | a. a standardized reconstruction pipeline has to be used when aggregating data. | Agreement | No consensus | 14/25 | 56% |
| 143 | b. it is preferred that a standardized reconstruction pipeline is used when aggregating data. | Agreement | Consensus | 25/25 | 100% |
| 3C-2: Best practice recommendation includes: | |  |  |  |  |
| 144 | a. Using denoising techniques to denoise 13C data. | Uncertain | No consensus | 11/24 | 46% |
| 145 | b. Sum-of-squares combination of multi-channel data. | Uncertain | No consensus | 4/20 | 20% |
| 146 | c. HP data-driven approaches for combination of multi-channel data. | Agreement | No consensus | 13/20 | 65% |
| 147 | d. Zero-filling prior to analysis. | Agreement | No consensus | 10/20 | 50% |
| 148 | e. Spatial filtering prior to analysis. | Uncertain | No consensus | 8/21 | 38% |
| 149 | f. Spectral filtering prior to analysis. | Agreement | No consensus | 12/21 | 57% |
| 150 | g. Spatial distortion correction (e.g. EPI displacement and ghosting artifact corrections, spiral off-resonance correction). | Agreement | Consensus | 22/23 | 96% |
| 3D-1: To facilitate analysis, all sites in a multi-center study have to record the following study parameters: | |  |  |  |  |
| 151 | a. ‘Standard’ scan parameters, such as TR, TE, FOV, matrix size, and bandwidth need to be reported by each site. | Agreement | Consensus | 25/25 | 100% |
| 152 | b. Timing parameters for the pyruvate injection and flush (start, duration) and acquisition (start : interval : end) need to be reported by each site. | Agreement | Consensus | 24/25 | 96% |
| 153 | c. If a spectrally-selective RF pulse is used, the frequency response specifications and frequency offsets for each metabolite need to be clearly stated by each site. | Agreement | Consensus | 24/25 | 96% |
| 154 | d. If a variable flip angle scheme is used, the flip angles for each metabolite and each timepoint need to be reported by each site. | Agreement | Consensus | 25/25 | 100% |
| 155 | e. If a multi-echo readout is used for chemical shift encoding, the echo-spacing, number of echoes, and metabolite frequencies used in the reconstruction should be reported by each site. | Agreement | Consensus | 25/25 | 100% |
| 4A-1: For multi-center studies: | |  |  |  |  |
| 156 | a. all sites have to store all 13C k-space raw data in addition to reconstructed images/spectra. | Agreement | No consensus | 17/26 | 65% |
| 157 | b. it is preferred that all sites have to store all 13C k-space raw data in addition to reconstructed images/spectra. | Agreement | Consensus | 26/26 | 100% |
| 4A-2: Viable data for storage includes: | |  |  |  |  |
| 158 | a. Raw data (k-space) | Agreement | Consensus | 25/25 | 100% |
| 159 | b. Minimally processed data (reconstructed images/spectra) | Agreement | Consensus | 25/25 | 100% |
| 160 | c. Processed data (parameter maps) | Agreement | Consensus | 24/25 | 96% |
| 4A-3: Preferred data for storage are: | |  |  |  |  |
| 161 | a. Raw data (k-space) | Agreement | Consensus | 25/25 | 100% |
| 162 | b. Minimally processed data (reconstructed images/spectra) | Agreement | Consensus | 24/25 | 96% |
| 163 | c. Processed data (parameter maps) | Agreement | Consensus | 23/25 | 92% |
| 4A-4: For multi-center studies, the following additional data has to be recorded: | |  |  |  |  |
| 164 | a. QC - pyruvate concentration | Agreement | Consensus | 25/28 | 89% |
| 165 | b. QC - EPA concentration | Agreement | Consensus | 23/28 | 82% |
| 166 | c. QC - Polarization | Agreement | No consensus | 17/28 | 61% |
| 167 | d. QC - pH | Agreement | Consensus | 24/28 | 86% |
| 168 | e. QC - temperature | Agreement | Consensus | 21/28 | 75% |
| 169 | f. Volume injected | Agreement | Consensus | 26/28 | 93% |
| 170 | g. Injection rate | Agreement | Consensus | 25/28 | 89% |
| 171 | h. Elapsed time from dissolution to start of injection | Agreement | Consensus | 23/28 | 82% |
| 172 | i. Elapsed time between start of injection and start of data acquisition | Agreement | Consensus | 24/28 | 86% |
| 4A-5: It is preferred that the following additional data are recorded: | |  |  |  |  |
| 173 | a. QC - pyruvate concentration | Agreement | Consensus | 27/28 | 96% |
| 174 | b. QC - EPA concentration | Agreement | Consensus | 26/28 | 93% |
| 175 | c. QC - Polarization | Agreement | Consensus | 26/28 | 93% |
| 176 | d. QC - pH | Agreement | Consensus | 27/28 | 96% |
| 177 | e. QC - temperature | Agreement | Consensus | 25/28 | 89% |
| 178 | f. Volume injected | Agreement | Consensus | 27/28 | 96% |
| 179 | g. Injection rate | Agreement | Consensus | 28/28 | 100% |
| 180 | h. Elapsed time from dissolution to start of injection | Agreement | Consensus | 28/28 | 100% |
| 181 | i. Elapsed time between start of injection and start of data acquisition | Agreement | Consensus | 28/28 | 100% |
| 4A-6: Basic patient metadata to be recorded/stored: | | *Question was removed* | | | |
| 182 | a. Diagnosis/disease |  |  |  |  |
| 183 | b. Disease stage (e.g. TNM status, grade category) |  |  |  |  |
| 184 | c. Age |  |  |  |  |
| 185 | d. Gender |  |  |  |  |
| 186 | e. Ethnicity |  |  |  |  |
| 187 | f. Weight and Height |  |  |  |  |
| 188 | g. Vital signs on day of study (e.g. blood pressure, heart rate) |  |  |  |  |
| 4B-1: For multi-center studies: | |  |  |  |  |
| 189 | a. data quality assessment has to be made. | Agreement | Consensus | 25/26 | 96% |
| 190 | b. it is preferred that data quality assessment to be made. | Agreement | Consensus | 25/26 | 96% |
| 4B-2: Viable method to assess data quality are: | |  |  |  |  |
| 191 | a. SNR Measurements | Agreement | Consensus | 26/26 | 100% |
| 192 | b. Assessment of artifact levels | Agreement | Consensus | 22/26 | 85% |
| 193 | c. Assessment of dynamic signal curves | Agreement | Consensus | 18/25 | 72% |
| 194 | d. External Reference-based assessments | Agreement | Consensus | 21/25 | 84% |
| 4B-3: Preferred method to assess data quality are: | |  |  |  |  |
| 195 | a. SNR Measurements | Agreement | Consensus | 26/26 | 100% |
| 196 | b. Assessment of artifact levels | Agreement | Consensus | 21/26 | 81% |
| 197 | c. Assessment of dynamic signal curves | Agreement | Consensus | 21/25 | 84% |
| 198 | d. External Reference-based assessments | Agreement | No consensus | 15/23 | 65% |
| 4B-4: Viable method for calculating 13C SNR for data quality assessment includes: | |  |  |  |  |
| 199 | a. Temporal peak Pyruvate SNR | Agreement | Consensus | 23/24 | 96% |
| 200 | b. Temporal AUC Pyruvate SNR | Agreement | Consensus | 23/24 | 96% |
| 201 | c. Temporal peak total Carbon SNR | Agreement | Consensus | 23/24 | 96% |
| 202 | d. Temporal AUC total Carbon SNR | Agreement | Consensus | 23/24 | 96% |
| 4B-5: Preferred method for calculating 13C SNR for data quality assessment are: | |  |  |  |  |
| 203 | a. Temporal peak Pyruvate SNR | Agreement | No consensus | 15/24 | 63% |
| 204 | 4b. Temporal AUC Pyruvate SNR | Agreement | Consensus | 19/24 | 79% |
| 205 | c. Temporal peak total Carbon SNR | Agreement | Consensus | 18/24 | 75% |
| 206 | d. Temporal AUC total Carbon SNR | Agreement | Consensus | 22/24 | 92% |
| 4C-1: For multi-center studies: | |  |  |  |  |
| 207 | a. HP MRI data has to be evaluated with quantitative metabolism metrics. | Agreement | No consensus | 13/26 | 50% |
| 208 | b. it is preferred that HP MRI data to be evaluated with quantitative metabolism metrics. | Agreement | Consensus | 25/26 | 96% |
| 4C-2: Viable options for evaluating HP MRI data include calculation of: | |  |  |  |  |
| 209 | a. Metabolite signals | Agreement | No consensus | 16/25 | 64% |
| 210 | b. Normalized Metabolite signals | Agreement | Consensus | 23/25 | 92% |
| 211 | c. Metabolite AUCs | Agreement | Consensus | 21/25 | 84% |
| 212 | d. Normalized Metabolite AUCs | Agreement | Consensus | 23/25 | 92% |
| 213 | e. Metabolite AUC ratios | Agreement | Consensus | 25/25 | 100% |
| 214 | f. Kinetic rates | Agreement | Consensus | 23/25 | 92% |
| 4C-3: The preferred metric for evaluating HP MRI data is: | |  |  |  |  |
| 215 | a. Metabolite signals | Agreement | No consensus | 13/26 | 50% |
| 216 | b. Normalized Metabolite signals | Agreement | Consensus | 18/26 | 69% |
| 217 | c. Metabolite AUCs | Agreement | Consensus | 17/25 | 68% |
| 218 | d. Normalized Metabolite AUCs | Agreement | Consensus | 23/25 | 92% |
| 219 | e. Metabolite AUC ratios | Agreement | Consensus | 24/25 | 96% |
| 220 | f. Kinetic rates | Agreement | Consensus | 19/25 | 76% |
| 4C-4: Multi-center HP MRI studies: | |  |  |  |  |
| 221 | a. have to incorporate a measurement of tissue perfusion to explore as a covariate with other metrics. | Uncertain | No consensus | 7/25 | 28% |
| 222 | b. it is preferred that multi-center HP MRI studies incorporate a measurement of tissue perfusion to explore as a covariate with other metrics. | Agreement | Consensus | 20/25 | 80% |
| 4D-1: For multi-center studies: | |  |  |  |  |
| 223 | a. an assessment of repeatability has to be made. | Agreement | Consensus | 24/26 | 92% |
| 224 | b. it is preferred that an assessment of repeatability to be made. | Agreement | Consensus | 26/26 | 100% |
| 4D-2: Viable options for assessing repeatability include use of: | |  |  |  |  |
| 225 | a. Static thermal phantoms | Agreement | Consensus | 22/25 | 88% |
| 226 | b. Dynamic phantoms (e.g. enzymatic conversion) | Agreement | No consensus | 16/25 | 64% |
| 227 | c. Same Subject injected with repeat HP 13C injections | Agreement | Consensus | 21/25 | 84% |
| 4D-3: Preferred options for assessing repeatability include use of: | |  |  |  |  |
| 228 | a. Static thermal phantoms | Agreement | Consensus | 20/25 | 80% |
| 229 | b. Dynamic phantoms (e.g. enzymatic conversion) | Agreement | No consensus | 14/25 | 56% |
| 230 | c. Same Subject injected with repeat HP 13C injections | Agreement | Consensus | 21/25 | 84% |
| 4D-4: For multi-center studies: | |  |  |  |  |
| 231 | a. an assessment of reproducibility has to be made. | Agreement | Consensus | 21/25 | 84% |
| 232 | b. it is preferred that an assessment of reproducibility to be made. | Agreement | Consensus | 25/25 | 100% |
| 4D-5: Viable options for assessing reproducibility include use of: | |  |  |  |  |
| 233 | a. Static thermal phantoms | Agreement | Consensus | 22/25 | 88% |
| 234 | b. Dynamic phantoms (e.g. enzymatic conversion) | Agreement | Consensus | 18/25 | 72% |
| 235 | c. Same Subject injected with HP 13C injections at more than one site | Agreement | Consensus | 20/25 | 80% |
| 4D-6: Preferred options for assessing reproducibility include use of: | |  |  |  |  |
| 236 | a. Static thermal phantoms | Agreement | Consensus | 18/25 | 72% |
| 237 | b. Dynamic phantoms (e.g. enzymatic conversion) | Agreement | No consensus | 14/25 | 56% |
| 238 | c. Same Subject injected with HP 13C injections at more than one site | Agreement | Consensus | 19/25 | 76% |
| 4E-1: Best practice for visualizing hyperpolarized MR data includes: | |  |  |  |  |
| 239 | a. Anatomical reference image required | Agreement | Consensus | 24/25 | 96% |
| 240 | b. Metabolite maps overlaid on anatomy | Agreement | Consensus | 23/25 | 92% |
| 241 | c. Metabolite maps next to anatomy | Agreement | No consensus | 16/25 | 64% |
| 242 | d. Images without masking to ROIs | Agreement | Consensus | 19/25 | 76% |
| 243 | e. Use of transparency in overlaid maps | Agreement | Consensus | 22/25 | 88% |
| 244 | f. Interpolation | Uncertain | No consensus | 11/25 | 44% |
| 4E-2: For multi-center studies: | |  |  |  |  |
| 245 | a. all sites have to use the same visualization methodology when evaluating data. | Agreement | Consensus | 19/25 | 76% |
| 246 | b. it is preferred that all sites to use the same visualization methodology when evaluating data. | Agreement | Consensus | 25/25 | 100% |

***Supplementary Table S3****: Summary of statements scoring for Round 1 and Round 2.*

| **Consensus round** | **Agreement**  **with consensus,**  *n* (%) | **Disagreement**  **with consensus,**  *n* (%) | **Uncertainty or**  **no consensus,**  *n* (%) |
| --- | --- | --- | --- |
| Round 1 (*n*=246) | 133 (54.1%) | 3 (1.2%) | 110 (44.7%) |
| Round 2 (*n*=235) | 146 (62.1%) | 11 (4.7%) | 74 (33.2%) |

***Supplementary Table S4****: Summary of statement additions, deletions, and changes made after round 1 and during round 2.*

| Altered statement post- round 2 | Previous statement | New statement |
| --- | --- | --- |
|  | 2A-5: Viable options for participation in multi-center study are: | Removed |
| 27 | a. 0.5T | Removed |
| 28 | b. 1.5T | Removed |
| 29 | c. 3T | Removed |
| 30 | d. 7T | Removed |
|  | 4A-4: For multi-center studies, the following additional data has to be **recorded**: | 4A-4: For multi-center studies, the following additional data has to be **recorded and reported**: |
| 164 | a. QC - pyruvate concentration | a. QC - pyruvate concentration |
| 165 | b. QC - EPA concentration | b. QC - EPA concentration |
| 166 | c. QC - Polarization | c. QC - Polarization |
| 167 | d. QC - pH | d. QC - pH |
| 168 | e. QC - temperature | e. QC - temperature |
| 169 | f. Volume injected | f. Volume injected |
| 170 | g. Injection rate | g. Injection rate |
| 171 | h. Elapsed time from dissolution to start of injection | h. Elapsed time from dissolution to start of injection |
| 172 | i. Elapsed time between start of injection and start of data acquisition | i. Elapsed time between start of injection and start of data acquisition |
|  | 4A-5: It is preferred that the following additional data are **recorded**: | 4A-5: It is preferred that the following additional data are **recorded and reported**: |
| 173 | a. QC - pyruvate concentration | a. QC - pyruvate concentration |
| 174 | b. QC - EPA concentration | b. QC - EPA concentration |
| 175 | c. QC - Polarization | c. QC - Polarization |
| 176 | d. QC - pH | d. QC - pH |
| 177 | e. QC - temperature | e. QC - temperature |
| 178 | f. Volume injected | f. Volume injected |
| 179 | g. Injection rate | g. Injection rate |
| 180 | h. Elapsed time from dissolution to start of injection | h. Elapsed time from dissolution to start of injection |
| 181 | i. Elapsed time between start of injection and start of data acquisition | i. Elapsed time between start of injection and start of data acquisition |
|  | 4A-6: Basic patient metadata to be recorded/stored: | Removed |
| 182 | a. Diagnosis/disease | Removed |
| 183 | b. Disease stage (e.g. TNM status, grade category) | Removed |
| 184 | c. Age | Removed |
| 185 | d. Gender | Removed |
| 186 | e. Ethnicity | Removed |
| 187 | f. Weight and Height | Removed |
| 188 | g. Vital signs on day of study (e.g. blood pressure, heart rate) | Removed |
